# Supplementary material for: Blockade of IL-10 Signaling Ensures Mifamurtide Efficacy in Metastatic Osteosarcoma
Source: Cancers (Basel). 2023 Sep 27;15(19):4744. doi: 10.3390/cancers15194744 (PMC10571595; doi:10.3390/cancers15194744)
Supplement: Supplementary file 1 [file cancers-15-04744-s001.zip › cancers-2581002-supplementary.pdf]

Figure S1 Supplementary

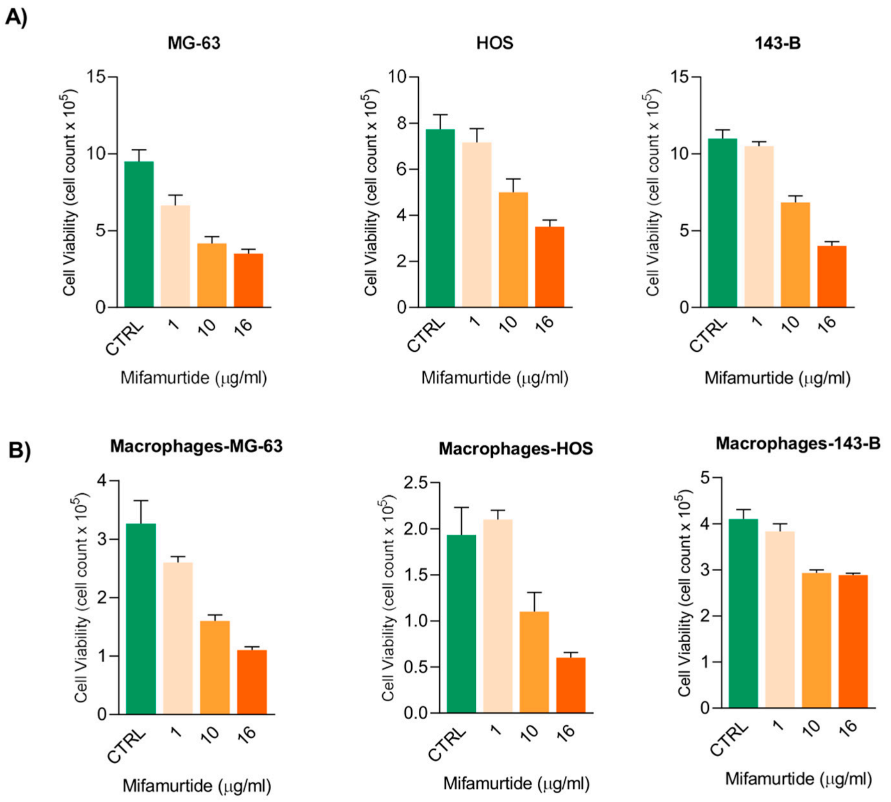

Fig.S1 Suppl. Concentration-related effects of mifamurtide on MG-63, HOS and 143-B cells and on macrophages viability (A, B). Assay was estimated by a FACS analysis after treatment with Mifamurtide (1, 10, 16 μg/ml). The results are presented as cell number per 10^5 (macrophages) and as the mean percentage ± SD.

Figure S2 Supplementary

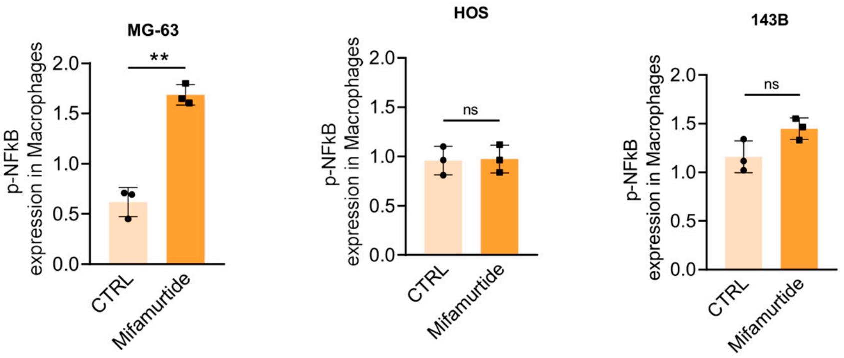

Fig. S**2 Suppl**. **Mifamurtide affects NF-kB pathways.** Mifamurtide triggers NF-kB signaling pathways only in macrophages co-cultured with MG-63 cells. Mifamurtide signaling on MG-63, HOS and 143-B OS cells in co-culture with macrophages after 24h of treatment. Quantification of p-NFkB/NFkB protein expression levels through AlphaLISA assay in macrophages co-cultured with OS cells. Significance was calculated by unpaired Student T-test analysis. *P < 0.05, **P < 0.01, ***P < 0.001 *vs* CTRL.

Figure S3 Supplementary

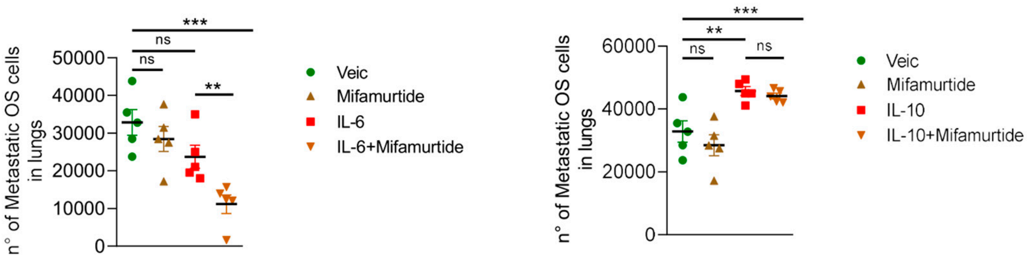

Fig. S3 Suppl. **The IL-6 and IL-10 administration, alone or with mifamurtide, affects lung metastasis dissemination in a different way**. BALB/c mice injected in tail vein with CellTracker green, fluorescent K7M2 OS murine cancer cells and treated with intraperitoneal injections of mifamurtide, IL-6 and IL-10 alone or combined with mifamurtide. After 24 hours, mice were sacrified, lungs isolated and dissociated, and cell suspension was then analyzed through flow cytometric analysis. Number of OS cells were then quantified.
